# Optimization of nutritional and sensory properties of fermented oat-based composite beverage

**DOI:** 10.1016/j.heliyon.2022.e10771

**Published:** 2022-09-30

**Authors:** Getaneh Firew Alemayehu, Sirawdink Fikreyesus Forsido, Yetenayet B. Tola, Endale Amare

**Affiliations:** a Department of Post-Harvest Management, Jimma University, Jimma, Ethiopia; b Department of Chemistry, Debre Markos University, Debre Markos, Ethiopia; c Food Science and Nutrition Research Directorate, Ethiopian Public Health Institute, Addis Ababa, Ethiopia

**Keywords:** Composite flour, Fermentation, Lupine, Stinging nettle, Oat-based beverage, Toasting

## Abstract

Oat (*Avena sativa*) is well-known for its nutritional value and health-promoting properties. There are only a few oat-based value-added products on the market in Ethiopia, and this study attempted to develop a new product that is both nutritionally enhanced and sensory acceptable, therefore, the objective of this study was to optimize the nutritional and sensory properties of a beverage made from oat, lupine (*Lupinus albus*), stinging nettle *(Urtica simensis*), and premix. D-optimal mixture experimental design was used to generate 11 runs applying the following constraints: 60–70% toasted oat, 10–25% roasted and soaked de-bittered white lupine, 5–15% boiled stinging nettle leaves, and 10% premix (flour of toasted black cardamom (2.8%), malted wheat (2.8%), pumpkin (2.6%), spiced chili peppers (1.1%), and table salt (0.7%). Statistical model evaluation and optimization were carried out using Minitab 19 software. The nutritional composition of the product was assessed, and results show that increasing the proportion of oat flour in the blend resulted in a significant (p < 0.05) increase in fat, carbohydrate, gross energy, and mineral contents (Fe, Zn). An increase in lupine flour increased crude protein, crude fiber, gross energy, phytate, tannin, oxalate, and antinutrient to mineral molar ratios. In contrast increased in stinging nettle leaf powder increased the ash and beta-carotene contents. Sensory of 11 composite sample beverages and control (90% oat plus 10% premix) were also carried out by 50 untrained panelists. Consequently, eight responses were optimized: protein, fat, Fe, Zn, beta-carotene, taste, appearance, and overall acceptability. The optimal blending ratio obtained was 70% oats, 11.3% lupine, 8.7% stinging nettle flour, and 10.0% premix. The study's findings suggested that the optimal combination of these traditionally processed ingredients in a beverage can be considered a valuable food with the potential to improve diet quality.

## 1 Introduction

Fermented foods and beverages are vital components of people's diets and are socially acceptable for entertainment, traditional practices, and religious purposes. Fermented foods are more nutritious than unfermented foods because the microorganisms hydrolyzes nutrients that are usually indigestible to humans and synthesize several B-complex vitamins and growth factors while growing in the medium (Gokavi et al., 2005). Globally, Every community has distinct fermented food and beverage culture representing the ethnicity's heritage and sociocultural aspects (Anagnostopoulos and Tsaltas, 2019).

Ethiopia is rich in cultural diversity, and thus a wide range of foods and beverages are produced and consumed by various ethnic groups (Ashenafi, 2006). In both rural and urban communities, a wide variety of fermented foods and beverages are made from different ingredients, with cereal-based being the most popular. Some of these include *tella, tej, borde, shamita*, *korefe, keneto, bukire, Cheka, Ogol, Booka* (Abegaz et al., 2002a; Bacha et al., 1999; Berhanu, 2016; Shewakena et al., 2017). Many of these fermented beverages are intended for consumption as alcoholic beverages. However, only a few beverages, such as *Borde* and *Shamita*, were consumed as meal replacements (Ashenafi, 2006). On the other hand, if these beverages are adequately produced and utilized, they can significantly contribute to the health and nutrition of the consumers (Abegaz et al., 2002b).

The rise of the functional foods market prompted the development of oat-based fermented beverages over the last 30 years (Angelov et al., 2018). The rising consumer demand for healthy drinks and value-added foods, and scientific discoveries about oats' nutritional composition are the drivers of the promotion of oat-based foods. The ancient concept of cereal-based fermented foods can be applied to develop new nutritionally enhanced oat-based fermented drinks as healthy, quick, and convenient meal replacement drinks.

There is enormous potential for the production of different indigenous colored oats, lupine (*Lupinus albus)*, and stinging nettle *(Urtica simensis*) in Ethiopia, particularly in Gojjam, North West Amhara region (CSA, 2020, Getaneh et al., 2021; Nigussie, 2012). The indigenous oats produced in this area are high in fat, fiber, starch, and energy while being low in anti-nutrient content, resulting in high mineral bioavailability (Alemayehu et al., 2021). Oats are also known for their high levels of β-glucans, soluble fiber, and bioactive phytochemicals. On the other hand, white lupine grain is widely produced in this region and is known to enrich protein contents when blended with cereals to alleviate protein-energy malnutrition. While stinging nettle (*Urtica simensis*) is an endemic plant to Ethiopia, it is a good source of vitamins, fiber, and minerals (Bayba et al., 2020). Keflie (2020) identified it as a potential source of vitamin E, while Tsegaye et al. (2009) and Belay and Wondimu (2019) reported stinging nettle as a functional food.

Despite potential crops, the area is one of the food insecure hotspots, and under-nutrition remains a major public health concern (Zeray et al., 2019). According to Motbainor et al. (2015), the prevalence of stunting, underweight, and wasting in the East Gojjam zone was 37.5%, 22.0%, and 17.1%, respectively, while in the West Gojjam zone, the prevalence was 38.3% stunting, 22.5% underweight, and 18.6% wasting. Nutrient-dense formulated foods are extremely expensive and difficult to find in local markets. Cereals combined with other locally available nutrient-dense foods, on the other hand, may aid in the fight against malnutrition. The current research aims to develop nutrient-rich meal replacement beverage with improved sensory acceptability by utilizing an underutilized and relatively low-cost cereal-legume-vegetable combination. Toasted oat, roasted-soaked lupine, and boiled and dried stinging nettle leaves flours are the optimized base ingredients. Premixes such as cardamom, spiced chili peppers, and table salt were chosen to improve the sensory attributes. Malted wheat was used to start the fermentation of the blended flours, and pumpkin pulp flour was added to boost the product's beta-carotene content. There has been a lack of research on oat-lupine-stinging nettle blending in beverages. The objective of this study was thus to develop a nutritionally enhanced and acceptable fermented beverage from oat, lupine, stinging nettle, and premix. The study's findings could help commercial beverage manufacturers and households produce low-cost meal replacement beverages by utilizing locally available and underutilized plant food sources.

## 2 Materials and methods

### 2.1 Sample collection

The primary ingredients for making the beverage were oat, lupine, and stinging nettle, all of which were collected from the Gozamin District of East Gojjam, Ethiopia. Crop and vegetable experts from the East Gojjam Agricultural Office performed botanical identification of the base ingredients. The same authors investigated five oat varieties' nutritional composition (Alemayehu et al., 2021). Three were local varieties with distinct colors (black, white, and yellow), and two were newly adapted improved varieties. In this study, one of the local varieties, the black-colored one, was chosen over the others due to its superior nutritional quality and phytoconstituents (Alemayehu et al., 2021). White lupine variety with an accession number AC.26634 was used which was collected from the Adet Agricultural Research Center. Following the existing practice of using wild stinging nettle for food by the local community in the study area, it was collected and used as an ingredient. Black cardamom, malted wheat, pumpkin, spiced chili peppers, and table salt were used as ingredients for the premix. The ingredients, spiced chili peppers and table salt were purchased from a supermarket in Debre Markos city, while others were collected from Gozamin District and processed as described in section 2.2.

### 2.2 Sample preparation

#### 2.2.1 Oat flour preparation

The oat grains were toasted uniformly using a metal pan with pre-cleaned sand to protect oat products from enzymatic deterioration, ensure storage stability, and improve sensory properties, then de-hulled to remove the husk. The sand was used to ensure that the toasting was uniform, and the extent of heat treatment during toasting was controlled to give light brown oat grains. The toasted oat was milled into flour and sieved through a 0.5 mm sieve.

#### 2.2.2 Lupine flour preparation

Lupine grain was first processed to remove bitterness and de-hulled before being pulverized into flour using the method described in Getachew et al. (2012). Briefly, the cleaned lupine was roasted for 10 min in a metal pan with pre-cleaned sand. After cooling to room temperature, the roasted lupine grain was soaked in a bucket in a 1:10 ratio of tap water. The soaking water was changed twice a day until the bitterness of the lupine was removed, and this process was continued for eight days. The lupine was de-hulled and sun-dried before grinding in a laboratory mill and sieving through a 0.5 mm sieve.

#### 2.2.3 Stinging nettle flour preparation

To obtain stinging nettle leaf powder, a traditional processing method was used. Briefly, the soft and shoot parts of stinging nettle leaves were placed on a rattan cultural tray and gently massaged on a traditional sieve to remove the prickles. The smooth stinging nettle leaves were cleaned and washed in water to remove any foreign or extraneous substances before being boiled (until tender) and sun-dried. The dried leaves were milled in a laboratory miller and sieved through a 0.5 mm sieve.

The beverage's base ingredients (oat, lupine, and stinging nettle) were then mixed in various ratios (Table1) to produce 11 composite sample flours, which account for 90% of the total flours used in the beverages.

**Table 1:** The percentage ratio and actual weight (gram) of the ingredients in 11 blended beverages and control.

Run	Components (%)				Run	Components (g)			
	Oat	Lupine	Stinging nettle	Premix		Oat	Lupine	Stinging nettle	Premix
1	65	10	15	10	1	115.6	17.8	26.7	17.8
2	70	15	5	10	2	124.5	26.7	8.9	17.8
3	62.5	15	12.5	10	3	111.1	26.7	22.2	17.8
4	65	15	10	10	4	115.6	26.7	17.8	17.8
5	62.5	20	7.5	10	5	111.1	35.6	13.3	17.8
6	60	15	15	10	6	106.7	26.7	26.7	17.8
7	65	12.5	12.5	10	7	115.6	22.2	22.2	17.8
8	60	25	5	10	8	106.7	44.5	8.9	17.8
9	70	10	10	10	9	124.5	17.8	17.8	17.8
10	67.5	12.5	10	10	10	120.0	22.2	17.8	17.8
11	67.5	15	7.5	10	11	120.0	26.7	13.3	17.8
Control	90% oat (control) 10				Control	160.0	0	0	17.8

#### 2.2.4 Premix preparation

The premix components were prepared in the way described below. The malted wheat was made using the methods described in Bacha et al. (1999). Briefly, wheat grain was cleaned to remove dirt and extraneous materials and steeped in clean water for about a day. Then, excess water was strained-off and allowed to germinate for five days, covering it with *gulo* (*Ricinus communis* L*)* leaves. The malted wheat was sun-dried before milling in a laboratory mill and passing through a 0.5 mm sieve.

Pumpkin pulp powder was made using the method described in Dhiman et al. (2018). Pumpkin was thoroughly washed with tap water to remove physical dirt and soil before peeling with a stainless steel knife, re-washed, and cut into four quarters. The quarters were sliced into small pieces and dried at 65 °C for 10 h. The dried pieces were then powdered using a laboratory milling machine and then sieved through a 0.5 mm sieve.

Black cardamom seeds were lightly toasted in a metal pan, milled into powder, and sieved through a 0.5 mm sieve. Spiced chili peppers and powder salt were purchased at a supermarket in Deber Markos City. Spiced chili pepper is a hot and spicy condiment made from chili peppers and various spices. Black cardamom, spiced chili peppers*,* and table salt were primarily added to the beverage as a premix to impart characteristic flavor to the product (Bacha et al., 1999). The total premix made up 10% of the total flours used in the beverages.

### 2.3 Beverage formulation and preparation

The beverages were made in the way described in Bacha et al. (1999) with some modifications. Bacha et al. (1999) utilized barley and linseed as base ingredients. However, oat, lupine, and stinging nettle flours were used as base ingredients in this study. Some ingredients like pumpkin were also added to the formulation to increase the β-carotene content of this meal-replacing beverage. Eleven composite beverage samples were made from oat, lupine, stinging nettle, and premix flours. Natural fermentation was conducted using the method described in Ibrahim et al. (2005). In brief, 1000 g composite flour (base ingredient) was mixed with 157.2 g premix (31.3 g of roasted black cardamom flour, 31.3 g of malted wheat flour, 28.1 g of pumpkin powder) and 1350 mL water in a mixing bowl until a homogenized gruel-like paste was obtained. The homogenized composite dough was then fermented for 24 h at room temperature (22 ± 2 °C). Then the rest two premixes (12.5 g spiced chili pepper flour and 8.1 g table salt) were also added to the fermented beverage immediately before consumption. Sensory analysis was performed on the product while it was fresh. At the same time, nutrient and anti-nutrient analyses were carried out after flattening the sample on the tray and dehydrating it in an oven (Blast Air Oven, DHG-9240A, China) for 20 h at 55 °C, pulverized, and sieved with a mesh size of 0.5 mm.

Natural fermentation was used as a major processing method in this study as it results in a desirable biochemical modification of the food matrix caused by microorganisms and enzymes (Nkhata et al., 2018). It is one of the oldest forms of food processing and preservation technology. It enhances nutrient bioavailability, protein digestibility, essential amino acids, essential fatty acids, and vitamin absorption (Anal, 2019). Furthermore, fermenting cereals increases their shelf life, sensory properties, and nutritional value.

### 2.4 Experimental design and treatments

The Minitab® Version 19.0 software was used to generate 11 run constrained D-optimal mixture experiments. The constraints used were 60–70 g/100 g for oats, 10–25 g/100 g for lupine, and 5–15 g/100 g for stinging nettle. These ranges were based on previous similar works (Izuwa, 2021; Keyata et al., 2021). Ingredient food composition data was also used to determine the upper and lower constraints (Alemayehu et al., 2021; Bayba et al., 2020; Getachew et al., 2012). The oats, lupine, and stinging nettle proportions were converted to 90 g/100 g, with the remaining 10 g/100g set aside for the premix. The response variables from the 11 formulations were also analyzed using the same software (Minitab®, Version 19).

### 2.5 Analyses of nutrients

#### 2.5.1 Analysis of proximate composition

The beverage's proximate composition was determined following AOAC (2000). The moisture, crude protein, crude fiber, crude fat, and ash were determined using the Association of Official Analytical Chemists method numbers 925.10, 979.09, 962.09, 920.39, and 923.03, respectively. Total carbohydrate content was computed by summing the percentages of all proximate values (crude protein + moisture + ash + crude fat) and subtracting from 100. The gross energy values (kcal) of the beverage were calculated by multiplying the crude protein, fat, carbohydrate, and fiber values by Atwater's conversion factors, which were 4 kcal/g for protein, 9 kcal/g for fat, 4 kcal/g for carbohydrates, and 2 kcal/g for fiber (FAO, 2003).

#### 2.5.2 Analysis of minerals

Minerals such as iron, calcium, and zinc were analyzed using Atomic Absorption Spectrophotometer (AAS) (SHIMADZU, AA-6880F, Japan) following Jorhem (2000) method. Zinc, iron, and calcium are major limiting nutrients that are currently regarded as major nutrients and public health concerns in Ethiopia (EPHI, 2016, Umeta et al., 2005).

#### 2.5.3 Analysis of beta-carotene

Beta-carotene was extracted using the method developed by the Association of Official Analytical Chemists AOAC (1990), and it was determined spectrophotometrically (JASCO V-630, Japan) using the method described in Mustapha and Babura (2009).

### 2.6 Analysis of anti-nutrients

Latta and Eskin (1980) method was used to determine the phytate content. Tannin was determined using the Burns (1971) method, and the total oxalate content was determined using the AOAC (2000) method 974.24.

### 2.7 Estimation of mineral bioavailability

The antinutrient-to-mineral molar ratio was calculated by dividing the mole of anti-nutrients by the mole minerals (Nwajagu et al., 2021). The following molar ratios were calculated; (phytate: Ca): mg of phytate/molecular weight of phytate (660.04 g/mol): mg of calcium/atomic weight of calcium (40.08 g/mol); (Phy: Fe): mg of phytate/molecular weight of phytate: mg of iron/atomic weight of iron (55.8 g/mol); (Oxalate: Ca): mg of oxalates/molecular weight of oxalate (88 g/mol): mg of calcium/atomic weight of calcium; (Phy: Zn): mg of phytates/molecular weight of phytates: mg of zinc/atomic weight of zinc (65.38 g/mol); (Phy∗Ca: Zn): (mg of calcium/atomic weight of calcium)∗(mg of phytates/molecular weight of phytates)/(mg of zinc/atomic weight of zinc). The obtained molar ratios were then compared to the limiting ratios of phytate: Ca less than 0.24, phytate: Fe less than 1, phytate: Zn less than 15, oxalate: Ca less than 1, and phytate∗Ca: Zn less than 200 (Castro-Alba et al., 2019).

### 2.8 Sensory analyses

The sensory evaluations of the product were carried out by 50 untrained panelists from Debre Markos town, Ethiopia. The informed sensory consent was obtained from all participants. They were asked to rate the products' sensory attributes, such as visual appearance, flavor, taste, aroma, consistency, and overall acceptability. A five-point hedonic scale was used, with 1 indicating extreme dislike, 3 indicating neither like nor dislike, and 5 showing extreme liking (Meilgaard et al., 2006). Just before the test session, the panelists were given a brief description of the sensory evaluation procedure. The coded beverages were then served in random order to the panelists in glass cups.

### 2.9 Statistical analyses

Minitab®, Version 19, was used to analyze the responses from the 11 formulations (Table 2). As model terms, mixture components were considered, and mixture regression was chosen as a model-fitting method. The association between a response and a term was significant when the p-value was less than 0.05. Positive coefficients for interaction terms show that the term's components act synergistically. Whereas negative coefficients show that the term's components act antagonistically (Forsido et al., 2019). The researcher established lower and upper response goals, and the "sweet spot" was obtained graphically from the contour plot of the optimized responses (Montgomery, 2013).

**Table 2:** Proximate composition, calorific value, minerals, beta-carotene contents of oat-based beverages (dry base).

Run	Components (%)				Proximate (g/100 g DW)						Energy (kcal/100 g DW)	Minerals (mg/100 g DW)			β-car (μg/g DW)
	Oat	Lup.	S.N	Prem.	Moisture	Protein	Fat	Fiber	Ash	Total CHO		Ca	Fe	Zn	
1	65	10	15	10	9.5 ± 0.10	16.6 ± 0.53	7.5 ± 0.27	3.8 ± 0.10	3.3 ± 0.10	72.6 ± 1.75	416.7 ± 1.22	52 ± 1.00	3.7 ± 0.10	3.5 ± 0.17	11.5 ± 0.10
2	70	15	5	10	10.5 ± 0.10	19.9 ± 0.46	10.1 ± 0.53	3.6 ± 0.27	3.1 ± 0.17	66.9 ± 0.30	430.9 ± 3.61	55 ± 2.65	4.2 ± 0.10	4.2 ± 0.27	8.0 ± 0.20
3	62.5	15	12.5	10	9.4 ± 0.10	18.8 ± 0.52	8.9 ± 0.27	3.3 ± 0.17	3.2 ± 0.10	69.1 ± 0.66	425.1 ± 1.47	47 ± 1.73	3.8 ± 0.10	3.7 ± 0.10	11.1 ± 0.35
4	65	15	10	10	9.7 ± 0.17	19.4 ± 0.46	10 ± 0.70	3.2 ± 0.56	3 ± 0.17	67.6 ± 1.18	431.6 ± 3.29	48 ± 1.32	3.8 ± 0.10	3.8 ± 0.10	10.8 ± 0.30
5	62.5	20	7.5	10	10.2 ± 0.10	20 ± 0.46	9.6 ± 0.20	3.9 ± 0.27	3 ± 0.20	67.4 ± 0.27	428.2 ± 2.31	48 ± 0.87	3.8 ± 0.10	3.7 ± 0.17	9.5 ± 0.10
6	60	15	15	10	10 ± 0.62	18.7 ± 0.56	8.4 ± 0.56	3.9 ± 0.20	3.4 ± 0.36	69.5 ± 1.18	420.6 ± 2.86	48.5 ± 0.61	3.6 ± 0.10	3.4 ± 0.17	11.4 ± 0.70
7	65	12.5	12.5	10	9.5 ± 0.17	18.1 ± 0.80	9.7 ± 0.10	3.1 ± 0.10	3.2 ± 0.10	69 ± 0.90	429.5 ± 0.82	48 ± 0.35	4.1 ± 0.35	3.9 ± 0.10	11.2 ± 0.17
8	60	25	5	10	9.5 ± 0.10	20.2 ± 0.92	8.8 ± 0.10	3.9 ± 0.27	3 ± 0.17	68 ± 1.32	424.2 ± 0.87	45 ± 0.20	3.8 ± 0.10	3.5 ± 0.17	7.5 ± 0.17
9	70	10	10	10	10.4 ± 0.17	17.2 ± 0.27	9.9 ± 0.36	2.8 ± 0.10	3.1 ± 0.30	69.8 ± 0.36	431.5 ± 3.06	55 ± 0.35	5 ± 0.44	4.4 ± 0.10	11 ± 0.17
10	67.5	12.5	10	10	10 ± 0.20	18 ± 0.56	9.8 ± 0.30	2.9 ± 0.10	2.9 ± 0.17	69.3 ± 0.60	431.6 ± 0.82	51 ± 3.47	4 ± 0.36	4.1 ± 0.10	10.6 ± 0.17
11	67.5	15	7.5	10	10 ± 0.35	19.4 ± 0.36	9.8 ± 0.10	2.9 ± 0.27	2.9 ± 0.00	67.9 ± 0.44	431.6 ± 0.36	51 ± 0.44	4 ± 0.17	4.1 ± 0.27	9.2 ± 0.17
	90% oat (control) 10				9.8 ± 0.44	14.8 ± 0.56	10.2 ± 0.17	2.2 ± 0.10	1.8 ± 0.27	73.2 ± 0.35	439.4 ± 1.95	40 ± 0.95	3.3 ± 0.10	2.5 ± 0.27	7.3 ± 0.17

Values are means ± standard deviation of 3 analyses. The sum of oats, lupine, and stinging nettle in a run amounted to 90%, and 10% was the premix in all the runs. Lup: lupine, S.N: stinging nettle, Prem: premix, CHO: carbohydrate; β-car: β-carotene.

## 3 Results and discussion

Table 2 shows the nutritional composition of the oat-lupine-stinging nettle composite beverages; the proximate, mineral (calcium, iron, and zinc), and beta-carotene contents, and Table 3 shows the p-values for all of the responses, including sensory properties. Protein, fat, carbohydrate, gross energy, iron, zinc, beta-carotene, taste, appearance, and overall acceptability were all statistically significant (p < 0.05), furthermore, quadratic models fit the data well (R^2^ > 0.80). Fiber, ash, aroma, and consistency, on the other hand, were not statistically significant (p > 0.05). The linear models also fit the data better (p < 0.05) for protein, ash, gross energy, calcium, iron, zinc, beta-carotene, taste, and appearance. However, moisture, fiber, aroma, mouthfeel, and consistency did not fit well in either the quadratic or linear models.

**Table 3:** Analysis of variance p-value of nutritional composition and sensory properties of the beverage prepared from blends of oat, lupine, and stinging nettle.

Regression Model	Proximate						Energy	Minerals			β-Carotene
	Moisture	Protein	Fat	Fiber	Ash	Total CHO		Ca	Fe	Zn	β-car
Linear^a^	0.427	0.048	0.074	0.072	0.036	0.298	0.02	0.012	0.024	0.026	0.01
Quadratic^a^	0.237	0.003	0.043	0.125	0.082	0.200	0.019	0.011	0.036	0.028	0.009
Oat∗Lupine	0.19	0.035	0.796	0.604	0.327	0.479	0.823	0.13	0.01	0.036	0.425
Oat∗Stinging nettle	0.144	0.347	0.113	0.066	0.018	0.714	0.028	0.005	0.851	0.233	0.005
Lupine∗Stinging nettle	0.419	0.055	0.011	0.044	0.117	0.021	0.005	0.008	0.088	0.006	0.003
*R*^2^ (adjusted)	0.47	0.97	0.80	0.64	0.76	0.88	0.86	0.88	0.82	0.95	0.98

CHO: carbohydrate; β-car: β-carotene. ^*a*^ Model fitting method used is mixture regression. Regression p-value ≤ 0.05 indicates the model explains variation in the response.

### 3.1 Nutritional quality

#### 3.1.1 Proximate composition and energy contents

The beverages' protein content ranged from 16.6 to 20.2 g/100 g DW, and beverage 8 had the highest protein content, while beverage 1 had the least (Table 2). A significant (p < 0.05) relationship was found between the linear terms, oat and lupine blend, and protein content (Table 3). The protein content of composite beverages increased by 12.2–36.5% compared to the control (90% toasted-fermented oat plus 10% premix). It increased as the proportion of lupine flours in the blend increased, which could be attributed to lupine's high-protein content (30–40%) (Janusz, 2017). Combining cereals and legumes generally improves overall essential amino acid balance, which helps to address global protein malnutrition issues (Sibt-E-Abbas et al., 2015). According to Boukid et al. (2019), lupine has a high concentration of most amino acids (Arg, Asp, Glu, Gly, His, Ile, Leu, Phe, Pro, Ser, Thr, Tyr, and Val). Although lupine is rarely used in beverage applications, numerous studies indicate its use in bakery products for protein enhancement. Yegrem et al. (2021) reported a significant increase in the protein content of *injera* from 10.2% to 15.5% by combining tef flour (control) with 10% lupine flour, while, Yaver and Bilgiçli (2021) used 20% lupine flour in bread production and increased the protein content by 1.5-fold more compared to bread without lupine. Therefore, the high protein content of the beverages due to the addition of lupine to the blend could be beneficial to those who are protein deficient, particularly in developing countries.

Natural fermentation may also increase the protein content of a beverage due to the accumulation of microbial biomass or the concentration of protein already in the substrate, as carbohydrates are consumed during fermentation. Tian et al. (2010) for oats, Osman (2011) for pearl millet, and Nelofer (2018) for barley demonstrated an increase in protein after 24 h of natural fermentation. Some studies have found that fermentation increases not only the amount of crude protein but also the amount of amino acids. Natural fermentation of amaranth grain flours for 48 h increased the amount of almost all free amino acids except tyrosine, glutamic acid, and proline, as reported by Amare et al. (2015). On the other hand, Bocchi et al. (2021) reported that amino acids have the highest bio-accessibilities in fermented beverages.

Another important aspect of fermentation is the enhancement of protein digestibility in cereal and legume products (Nkhata et al., 2018). The partial breakdown of complex storage protein into more soluble forms improves protein digestibility. Plant protein is less digestible than animal protein. Poor protein digestibility can cause gastrointestinal upset, leading to protein excretion in the feces (Untersmayr and Jensen-Jarolim, 2008). In general, fermentation combined with other processing methods has more advantages than its unfermented counterparts.

The fat content of the beverages ranged from 7.5 to 10.1 g/100 g DW, which showed statistically significant differences (p < 0.05). The highest fat content was found in beverage 2, while the lowest was in beverage 1 (Table 2). A significant (p < 0.05) relationship was found between the linear terms, lupine and stinging nettle blend, and the fat content (Table 3). When compared to the control, the fat content of composite beverages decreased by 1–26%. The beverages' fat content was directly related to the percentage of oat flour in the blend, which could be due to oats' high-fat content compared to lupine and stinging nettle. According to Alemayehu et al. (2021), the fat content of five Ethiopian oat samples, both native and improved, ranged from 6.7 to 10.5, which is very high compared to other cereals. Oats are also well-known for being a good source of high-quality unsaturated lipids (Angelov et al., 2018). Some research works indicated the use of oats to make various fermented and non-fermented beverages (Bocchi et al., 2021). However, much of the research efforts were aimed at increasing the beta-glucan and soluble dietary fiber content of the final beverage. On the other hand, some studies used oats to increase the fat content of the final product. Chauhan et al. (2018) reported that incorporating 25% oat flour increased the fat content of noodles by 3.28% when compared to the control (100% wheat noodles).

The ash content of the beverages ranged between 2.9 and 3.4 g/100 g DW, and there were no significant changes observed among the sample beverages. However, the ash content was found to have a statistically significant (p < 0.05) relationship with the oat and stinging nettle blend (Table 3). Beverage 6 had the highest ash content, while beverages 10 and 11 had the lowest (Table 2). Comparing composite beverages to the control, the ash content was increased by 61.1–88.9%. It showed a direct relationship with the proportion of stinging nettle powder in the blend, which could be because of the high ash content in the stinging nettle than other components. According to Bayba et al. (2020), the ash content of stinging nettle was 17.2–24.3%, which was found to be the highest of most common vegetables. The highest ash content suggests that stinging nettle is rich in minerals.

The fiber content of the beverages ranged from 2.8 to 3.9 g/100 g DW, and no significant changes were observed among the sample beverages. Different flour ratio combinations yielded the highest fiber content (beverages 5, 6, and 8), while beverage 9 yielded the lowest fiber content (Table 2). A significant (p < 0.05) relationship was found between the fiber content and the blend of lupine and stinging nettle (Table 3). When compared to the control, the fiber content of composite beverages increased by 27.2–77.2%. The obtained result showed that the crude fiber contents increased as the proportion of lupine flour in the blend increased. Lupine is rich in dietary fiber, Getachew et al. (2012) reported 11% of crude fiber for lupines collected from Dangilla and Chagni, Ethiopia. Yaver and Bilgiçli (2021) used 20% lupine flour in bread production and increased the fiber content by 2.5-fold compared to bread without lupine.

The beverages' total carbohydrate content ranged from 66.9 to 73.2 g/100 g DW, and significant variation was observed among the sample beverages. Beverage 1 had the highest total carbohydrate content, whereas beverage 2 had the lowest (Table 2). Compared to the control, the total carbohydrate content of composite beverages was decreased by 0.82–8.6%. The carbohydrate content of the drinks increased as the proportion of oat flour in the blend increased. This is due to the fact that cereals have higher carbohydrate content than legumes and vegetables. According to Alemayehu et al. (2021), the carbohydrate content of oats was 73.3 g/100 g DW, while lupine had a carbohydrate content of 38.9 g/100 g DW (Getachew et al., 2012). According to Gokavi et al. (2005), oat-based beverages contain a high proportion of desirable complex carbohydrates, which could reduce the risk of certain cancers and constipation. Das and Maria (2017) also reported fermented oats as a functional food with high carbohydrate content and health-promoting constituents that could reduce the risk of various metabolic disorders.

The beverages' energy content ranged from 416.7 to 431.6 kcal/100 g DW, and significant variation was recorded among sample beverages. Beverages 10 and 11 had the highest gross energy value, while beverage 1 had the lowest gross-energy value (Table 2). The gross-energy values of composite beverages decreased by 1.8–5.1% when compared to the control. The high-energy beverage was made from a formulation that contains a high proportion of oat and lupine flours. It could be due to oats' high carbohydrate and fat content and lupine's high protein content. The blend of oats and stinging nettle, lupine and stinging nettle, and the gross energy values have a statistically significant (p < 0.05) relationship. This study disagreed with the findings of Yegrem et al. (2021), who showed the increments of calorific energy in *injera* from 393.8 to 400.4 kcal/100 g upon 20% lupine flour addition with tef flour. Onilude et al. (1999) found higher gross energy (462.6–508 kcal/100 g) of weaning food, made from composite blends of cereals and legumes, prepared similarly to this study (fermented and toasted). Oats had the highest oil content of any cereal, coming in close to oil crops, which would eventually contribute significantly to the high calorific energy values of the product (Liu and Wise, 2021). Furthermore, the malt in the premix also boosts the fermented beverages' gross energy. According to Forsido et al. (2020), fermenting cereal flours for 24 h with 2% malt increased the energy density. The energy contributions of macronutrients in this study were almost within the range of FAO/WHO (2003) recommendations for chronic disease prevention, protein (10–15% energy), total fat (15–30% energy), and total carbohydrate (55–75% energy) from a food product's total energy.

Most naturally fermented foods and artisanal beverages are naturally fermented, which means no control over the microbiota or substrate used (Ashenafi, 2006). The consortia could be comprised of various bacteria, fungi, and yeasts (Tafere, 2015). Bacteria involved in natural fermentation include *Lactobacillus, Leuconostoc, and Pediococcus, specifically L. plantarum, L. brevis, L. paracasei, L. acidophilus, Lactobacillus casei, L. sanfranciscensis, L. pontis, L. alimentarius, L. fructivorans, L. reuteri, L. fermentum,* etc. (Leeuwendaal et al., 2022)*.* Yeast species involved in natural fermentation include *Saccharomyces cerevisiae* (the most common), *Saccharomyces uvarum, Brettanomyces anomalus, Candida javanica, Geotrichum candidum, Hansenula anomala, Pichia burtonii, Rhodotorula glutinis, Saccharomycopsis fibuligera, Saccharomyces dairensis, Saccharomyces globosus, Saccharomyces kluyveri, Saccharomyces sake, Torulopsis versatilis, Trichosporon pullulans, Zygosaccharomyces rouxii sake* etc (Leeuwendaal et al., 2022; Tamang and Fleet, 2009). Even though natural fermentations are not standardized, they are a valuable source of bioactive compounds such as antioxidants, bioactive beeps, short-chain fatty acids, amino acids, vitamins, and minerals (Cuvas-Limon et al., 2021). Antibiotics, antimicrobial peptides, carbon dioxide, alcohol, vitamins, folates, and organic acids are the primary and secondary metabolites that spontaneous fermentation produces (Cuvas-Limon et al., 2021).

Nowadays, fermented oat-based foods offer attractive prospects within the market of non-dairy functional products since they are suitable substrates for the delivery of probiotic microorganisms (Gupta and Bajaj, 2017). They have been tested as functional and probiotic foods due to their nutritive and health-promoting properties (Peyer et al., 2016; Marco et al., 2017). Bacteriocins and exopolysaccharides (EPS) are two bioactive compounds derived from fermented oats beverages. Bacteriocins are proteinaceous antimicrobial substances produced by some *Lactobacillus, Lactococcus, Pediococcus,* yeasts, and filamentous fungi strains (Nyanzi and Jooste, 2012). It inhibits the development of pathogens such as *E. coli, Salmonella typhi, Staphylococcus aureus, Bacillus cereus, Listeria monocytogenes,* and *Clostridium botulinum* (Das et al., 2017; Nyanzi and Jooste, 2012), Exopolysaccharides (EPS) are another beneficial component that imparts good flavor and texture characteristics to fermented foods. They are produced by fermenting Lactic Acid Bacteria (LAB), which predominate in the natural microflora of fermented foods (Castellone et al., 2021).

According to Mårtensson et al. (2005) and Marsh et al. (2014), oat-based beverages have natural prebiotic properties due to the presence of indigestible fibers and the presence of diacetyl acetic acid and other aromatic compounds, which make them more palatable and potentially cheaper to produce. Mårtensson et al. (2005) demonstrated that fermented oat beverages contain a high beta-glucan content and act as a prebiotic by increasing the number of *bifidobacteria* in the gut. Indeed, a fermented oat drink containing two strains of *Bifidobacterium longum* was shown to normalize bowel movements in elderly patients (Ouwehand et al., 2008). To fully benefit from and scale-up traditional natural fermentation processes, it is essential to understand better the microbiology and biochemistry behind these spontaneously fermented products.

#### 3.1.2 Mineral contents

Iron contents varied from 3.6 to 5 mg/100 g DW and showed a statistically significant (p < 0.05) change among the sample beverages. Beverage 9 had the highest Fe content, while beverage 6 had the lowest Fe content (Table 2). The iron content has a significant (p < 0.05) relationship with blends of oat and lupine. The iron content of composite beverages was increased by 2.5–37.5% when compared to the control. Stinging nettle had a very high ash content, which could provide a lot of minerals. However, beverages' iron content in this study was related to the oats' proportion in the blend. According to Zhang et al. (2007), incorporating oats made iron enrichment of beverages possible.

The zinc content of 11 beverage samples ranged from 3.4 to 4.4 mg/100 g DW and showed a statistically significant (p < 0.05) change among the sample beverages (Table 3). A significant (p < 0.05) relationship was found between the linear terms, oats and lupine blend, lupine and stinging nettle blend, and zinc content. Lupine and oats were antagonistic, whereas lupine and stinging nettle were synergistic. Beverage 9 had the highest Zn content, whereas beverage 6 had the lowest Zn (Table 2). The zinc content of composite beverages increased from 3 to 76% compared to the control. It increased when the proportion of oat flour in the composite increased, which could be due to oats' higher zinc content than the other ingredients. Özcan et al. (2017) reported a higher content of zinc in oats than in other whole grains.

The calcium content in the sample beverages ranged from 45 to 55 mg/100 g DW, with no statistically significant differences observed. However, a significant (p < 0.05) relationship was exhibited between the blend of oats and stinging nettle and the calcium content. The calcium content of beverages 2 and 9 was the highest, while beverage 8 was the lowest (Table 2). The calcium content of composite beverages increased from 12.5 to 37.5% and was related to the proportion of oat flour in the blend. According to Duta and Culetu (2015), the calcium content of the cookies increased from 109.3 to 161.3 mg/100 g after oat bran was added. Oat bran is a skin integral part of whole oats; because this study used whole oats, the increase in the calcium of the products with oats proportion could be due to oats high proportion (60–70%) in the blend and oats' high calcium content in its bran.

Natural fermentation, which was used in this study, also aids in the breakdown of mineral-antinutritional factor interactions, which is common in cereals. Natural fermentation in the blend may disrupt these interactions, allowing minerals to be liberated and absorbed in the body.

#### 3.1.3 β-carotene content

The β-carotene content of beverage samples ranged from 7.5 to 11.5 mg/100 g DW and showed statistically significant (p < 0.05) variation among the sample beverages. The β-carotene content and blends of oats and stinging nettle, lupine and stinging nettle had a statistically significant (p < 0.01) relationship. Both oats and stinging nettle and lupine and stinging nettle act synergistically. Beverage 1 had the highest β-carotene content, while beverage 8 had the lowest (Table 2). The beta-carotene content of composite beverages was increased by 2.7–57.5%. It was proportional to the stinging nettle flour in the blend, most likely due to the high β-carotene content in the stinging nettle.

#### 3.1.4 Anti-nutrient content

Table 4 presents the results of anti-nutrients and molar ratios of anti-nutrients to minerals for the formulated oat-based beverages. The phytate content of the sample beverages (163–173 mg/100 g DW) varied significantly (p < 0.05), and also the sample beverages had a significant (p < 0.05) relationship between the linear terms and the phytate content. Beverages 5 and 8 had the highest phytate content, whereas beverage 9 had the lowest phytate content. The phytate content of composite beverages was decreased by 12.4–17.4%, which was related to the proportion of lupine flour in the blend. This decline could be due to lupine having higher phytate content than oat and stinging nettle. According to Kasprowicz-Potocka et al. (2021), the phytate content of lupine (*Lupinus albus* and *Lupinus cosentinii*) ranged from 0.4 to 12 g/100 g. In comparison to many cereal grains, this was a relatively high amount. Phytate may reduce the intestinal absorption of several minerals by forming insoluble compounds in the intestinal tract (Buddrick et al., 2014). However, it is worth noting that phytate content in lupine can be significantly reduced by fermentation during the beverage-making process. This decrease could be attributed to the enzymatic degradation of phytate by fermenting microorganisms. The microorganisms in the natural flora of grains cause the fermented food matrix to lose by degrading the phytate (Nkhata et al., 2018). Amylase, pullulanase, phytase, and other glucosidases are enzymes that degrade antinutritional factors and break down complex macronutrients into simpler and more digestible forms. According to Buddrick et al. (2014) and Lopez et al. (2001), as fermentation time increased, the phytate content of wholegrain flour dough decreased. Khan et al. (2018) reported that after 24 h of fermentation, phytate levels in lupine were reduced by 29–50%. Adekanmi et al. (2009) indicated that soaking and toasting legumes (Tigernut, *Cyperus esculentus* L.) reduced phytate content by 27–44%. In the present study, ingredients used undergo processes that could cause to decrease in phytate. The soaking and roasting in lupine, toasting in oats, phytase from malt wheat in the premix, and fermentation of the composite flour contributes to the degradation of phytate. Even though there is no official intake limit for phytate/phytic acid, the phytate content of sample beverages in this study demonstrated a lower amount.

**Table 4:** Anti-nutrients and molar ratios of anti-nutrients to minerals of oat-based beverages.

Run	Components (%)				Antinutrients (mg/100 g DW)			Molar ratios of antinutrients to minerals				
	Oat	Lup.	S.N	Prem.	Phy	Tan	Ox	(Phy: Ca)^1^	(Ox: Ca)^2^	(Phy: Fe)^3^	(Phy: Zn)^4^	(Phy∗Ca: Zn)^5^
1	65	10	15	10	164 ± 2.65	17 ± 1.35	16 ± 0.66	0.19 ± 0.01	0.14 ± 0.01	3.75 ± 0.07	4.64 ± 0.22	6.02 ± 0.18
2	70	15	5	10	170 ± 1.37	21 ± 0.78	23 ± 0.35	0.19 0 ± 0.01	0.19 ± 0.01	3.42 ± 0.11	4.02 ± 0.27	5.51 ± 0.37
3	62.5	15	12.5	10	169 ± 1.00	21 ± 0.35	22 ± 0.70	0.22 ± 0.01	0.21 ± 0.01	3.76 ± 0.12	4.52 ± 0.10	5.31 ± 0.11
4	65	15	10	10	169 ± 2.02	20 ± 0.61	21 ± 1.73	0.21 ± 0.00	0.2 ± 0.01	3.76 ± 0.12	4.41 ± 0.08	5.28 ± 0.10
5	62.5	20	7.5	10	173 ± 0.82	23 ± 0.52	24 ± 0.46	0.22 ± 0.00	0.23 ± 0.01	3.85 ± 0.12	4.63 ± 0.19	5.55 ± 0.13
6	60	15	15	10	171 ± 0.82	22 ± 0.44	20 ± 0.17	0.21 ± 0.00	0.19 ± 0.00	4.02 ± 0.13	4.99 ± 0.23	6.04 ± 0.20
7	65	12.5	12.5	10	166 ± 1.51	19 ± 0.46	19 ± 0.53	0.21 ± 0.00	0.18 ± 0.00	3.42 ± 0.26	4.22 ± 0.12	5.05 ± 0.09
8	60	25	5	10	173 ± 1.23	24 ± 0.78	25 ± 1.25	0.23 ± 0.00	0.25 ± 0.01	3.85 ± 0.10	4.91 ± 0.22	5.5 ± 0.27
9	70	10	10	10	163 ± 0.36	19 ± 1.14	15 ± 0.40	0.18 ± 0.00	0.12 ± 0.00	2.77 ± 0.26	3.67 ± 0.08	5.04 ± 0.11
10	67.5	12.5	10	10	168 ± 0.92	19 ± 0.44	19 ± 0.46	0.2 ± 0.00	0.17 ± 0.01	3.57 ± 0.33	4.06 ± 0.09	5.16 ± 0.27
11	67.5	15	7.5	10	169 ± 0.36	20 ± 0.46	22 ± 0.17	0.2 ± 0.00	0.2 ± 0.00	3.58 ± 0.15	4.09 ± 0.25	5.21 ± 0.33
	90% oat (control)			10	197.4 ± 1.39	28.5 ± 0.46	31.4 ± 0.44	0.3 ± 0.01	0.36 ± 0.01	5.06 ± 0.13	7.89 ± 0.86	7.86 ± 0.81

Values are means ± standard deviation of 3 analyses. The sum of oats, lupine, and stinging nettle in a run amounted to 90%, and 10% was the premix in all the runs. Lup: lupine, S.N: stinging nettle, Prem: premix, Phy: phytate; Tan: tannin; Ox: Oxalate; (Phy: Ca)^1^: molar ratio of phytate to calcium; (Ox: Ca)^2^: molar ratio of oxalate to calcium; (Phy: Fe)^3^: molar ratio of phytate to iron; (Phy: Zn)^4^: molar ratio of phytate to zinc; (Phy∗Ca: Zn)^5^: mole of phytate∗mole of calcium/mole of zinc.

The blending ratios cause a significant change (p < 0.05) in the tannin content (17–24 mg/100 g DW) of the beverage. Oats and lupine, and oats and stinging nettle, act antagonistically, whereas lupine and stinging nettle act synergistically. The highest tannin content was found in beverage 8, while the lowest was found in beverage 1. Compared to the control, the tannin content of composite beverages' decreased by 15.8–40.4%. The tannin content increased with lupine content in the blend. According to Grela et al. (2017), the tannin content of three lupine varieties ranged from 0.26 to 0.50 g/100 g, which is high when compared to the tannin content of oats and stinging nettle. Tannins are polyphenolic compounds that can form stable complexes with proteins, minerals, and vitamins A and B_12_. As a result, nutrient digestibility and availability are reduced (Grela et al., 2017). However, the results of this study show that the tannin content in the sample beverages was significantly low, which could be due to the lowering effect of traditional processing methods (roasting and natural fermentation of lupine) and blending effects with toasted oats and stinging nettle. Adeyemo and Onilude (2013) found that fermentation with *Lactobacillus plantarum* reduced tannins in soybean from 1.93 to 0.12 mg/g. Chude et al. (2018) found that cooking and fermentation significantly reduced the tannin content of legumes (groundnut) from 4.72 to 2.08 mg/100 g. Adekanmi et al. (2009) found that soaking and toasting reduced the tannin content of legumes (Tigernut, *Cyperus esculentus* L.) by 15–61%. Thus, in our study, the combined effects of these three processing methods (soaking, roasting, and fermentation) could potentially reduce the tannin content in our sample beverages. Tannic acid has a daily intake limit of 560 mg for adults (male and female) Sandberg (2002). In this regard, the daily intake limit could only be surpassed if one consumed more than 2333 g DW per day of the formulated beverages in this study. Thus, the inhibitory effects of tannin in the sample beverages were negligible.

The oxalate content of the beverages ranged from 15 to 25 mg/100 g DW (Table 4), with a statistically significant (p < 0.05) difference among the sample beverages (Table 5). A significant (*p* < 0.01) association was observed between the oats and lupine and the oxalate content. The highest oxalate content was found in beverage 8, while the lowest was found in beverage 9. The oxalate content of composite beverages' decreased by 20.4–52.2%. It increased in the same way that the phytate and tannin content did as the lupine ratio in the blend increased. Andrade et al. (2015) reported the oxalate content of lupines between 1.2 and 490 mg/100 g. Chude et al. (2018) found that cooking and fermentation significantly reduced the oxalate content of legumes (groundnut) from 1.85 to 0.85 mg/100g. While, Adekanmi et al. (2009) reported that soaking and toasting reduced the oxalate content of legumes (Tigernut, *Cyperus esculentus* L.) by 57–77%. The results of this study revealed a lower oxalate content in the beverage, which could be due to the combined effects of traditional processing methods (soaking, roasting, and fermentation of lupine, toasting of oats, and boiling of stinging nettle). According to Savage et al. (2000), treatment processes such as soaking and cooking may cause tissue breakdown and increase the outflow of soluble oxalate, resulting in low accessible oxalate for intake in the digestive tract. Oxalate is the most common risk factor for kidney stone formation and is responsible for the main crystalline component of nephrolithiasis. Patients are advised to limit their daily oxalate intake to 40–50 mg (Mitchell et al., 2019). In this study, however, the oxalate content of all sample beverages was found to be below the limits.

**Table 5:** Analysis of variance p-value of antinutritional and the molar ratio of anti-nutrient to minerals of sample beverages prepared from blends of oat, lupine, stinging nettle, and premix.

Regression Model	Anti-nutrients			Molar ratio of Anti-nutrient to Minerals				
	Phytate	Tannin	Oxalate	(Phy:Ca)^1^	(Ox: Ca)^2^	(Phy: Fe)^3^	(Phy: Zn)^4^	(Phy∗Ca: Zn)^5^
Linear^a^	0.207	0.016	0.018	0.119	0.026	0.051	0.093	0.046
Quadratic^a^	0.021	0.005	0.006	0.108	0.011	0.068	0.089	0.089
Oat∗Lupine	0.222	0.008	0.008	0.172	0.008	0.024	0.077	0.657
Oat∗Stinging nettle	0.201	0.011	0.806	0.041	0.126	0.824	0.51	0.039
Lupine∗Stinging nettle	0.288	0.012	0.689	0.122	0.241	0.186	0.63	0.037
R^2^ (adjusted)	0.93	0.97	0.96	0.9	0.91	0.81	0.78	0.59

Notes: ^*a*^ Model fitting method used is mixture regression. (Phy: Ca)^1^: molar ratio of phytate to calcium; (Ox: Ca)^2^: molar ratio of oxalate to calcium; (Phy: Fe)^3^: molar ratio of phytate to iron; (Phy: Zn)^4^: molar ratio of phytate to zinc; (Phy∗Ca: Zn)^5^: mole of phytate∗mole of calcium/mole of zinc.

### 3.2 Estimation of mineral bioavailability

The antinutrient-to-mineral molar ratios are an important predictor of mineral bioavailability. Lower molar ratios indicate higher mineral bioavailability (Udomkun et al., 2019). There was no statistically significant variation in phytate: Ca ratios among the 11 sample beverages, but there was a significant association between oats and stinging nettle and the phytate: Ca ratios. Beverage 8 had the highest phytate: Ca ratio, while beverage 9 had the lowest (Table 4). Composite beverage phytate: Ca ratios decreased from 23.2 to 39.9%. The beverages' phytate: Ca molar ratios ranged from 0.18 to 0.23, with values less than 0.24 indicating good calcium bioavailability (Tufa et al., 2016). All sample beverages had phytate: Ca molar ratios greater than the threshold, predicting good calcium bioavailability.

The oxalate: Ca molar ratios varied from 0.12 to 0.25 with significant changes among the sample beverages. The association between the blend of oats and lupine and oxalate: Ca molar ratio was statistically significant. Oats and lupine, lupine and stinging nettle act synergistically. The highest oxalate: Ca molar ratio was in beverage 8, while the lowest was found in beverage 9 (Table 4). The oxalate: Ca ratios decreased from 29.9 to 66.4% compared to the control. Castro-Alba et al. (2019) reported the permissible level of oxalate: Ca molar ratio is one, indicating that Ca absorption could not be impaired in all sample beverages.

The phytate: Fe molar ratios ranged from 2.76 to 4.02, with no significant change among the beverage samples. A significant association was observed between oats and lupine and the phytate: Fe molar ratio. Beverage 6 had the highest molar ratio, while beverage 9 had the lowest (Table 4). Composite beverage phytate: Fe molar ratio decreased from 20.5 to 45.4% compared to the control. According to Castro-Alba et al. (2019), phytate suppresses iron bioavailability when the molar ratio of phytates: Fe is above one. In this regard, the phytate: Fe molar ratios of all formulated sample beverages were higher than the critical value, indicating that iron bioavailability was lower. Iron fortification and the use of nutrient absorption enhancers have been recommended to reduce the inhibition effects. For example, Singh and Prasad (2018) found that the addition of ascorbic acid increased the iron bioavailability of red kidney beans from 1.04 to 11.57%, green gram (whole moong) from 1.79 to 6.48%, and wholemeal bread from 2.44 to 8.76%. Moreover, Zlotkin et al. (2003) reported that home-fortification with iron sprinkles was a very successful method of alleviating iron deficiency.

The phytate: Zn molar ratio is thought to be a better predictor of zinc bioavailability than total dietary phytate levels (Tufa et al., 2016). The highest and lowest phytate: Zn molar ratios were found in beverages 6 and 9, respectively (Table 4). Zinc bioavailability is increased when the phytate: Zn molar ratio is less than five and low when greater than 15 (Castro-Alba et al., 2019). The phytate: Zn molar ratios decreased by 36.3–53.1%, and the molar ratios of all beverages (3.67–4.98) were less than 5, indicating that Zn bioavailability was unlikely to be inhibited. The lower phytate: Zn ratios in this study could be attributed to phytic acid depletion during fermentation. According to Hunt and Beiseigel (2009), zinc absorption was reduced by 25% when the phytate: Zn molar ratio was increased from 4 to 15.

The phytate∗Ca: Zn molar ratio ranged from 5.04 to 6.03, with no significant difference among the sample beverages. There was a significant association between oats and stinging nettle, lupine and stinging nettle, and phytate∗Ca: Zn molar ratio. Beverages 6 and 9 had the highest and lowest phytate∗Ca: Zn molar ratios, respectively (Table 4). Beverages' phytate∗Ca: Zn molar ratios decreased from 22.7 to 35.4% compared to the control. Because calcium can inhibit zinc absorption when phytate levels are high, the Ca∗phytate: Zn ratio predicts zinc availability better than the phytate: Zn ratio. If the Ca∗phytate: Zn ratio is greater than 200, there will be interferences with zinc availability Castro-Alba et al. (2019). Thus, all the studied sample beverages' calcium bioavailability was above the threshold level.

One of the reasons that the estimated mineral bioavailability of the beverages is higher than the limiting values could be due to the natural fermentation used for the composite beverages and other processing methods applied to each ingredient. Natural fermentation has been used as a daily processing method for dietary meals in developing countries, and it fortunately, aids in breaking the interactions of minerals with antinutritional factors. Because of these desirable advantages, it has been regarded as an effective method of reducing the risk of mineral deficiency among populations in Ethiopia, particularly in developing countries in general.

### 3.3 Sensory quality

The sensory scores of beverages made from fermented composite flour of oats, lupine, stinging nettle, and premix are summarized in Table 6. The mean taste values for the beverages were in the range of 3.6–4.5, and the results showed a statistically significant variation. There was also a significant difference (p < 0.05) between the linear terms, oat and lupine, and the taste score. Beverages 8 and 9 had the highest mean taste scores, while beverages 5 and 6 had the lowest mean taste scores. When the ratios of oats and lupine in the blend were high, the taste score had high values. It could be due to oats' high-fat content, which consequently gives a palatable taste. The other reason could also be the removal of the bitter taste of lupine, which was brought by its high content of alkaloids, during sample preparation (roasting and soaking) (Alemu et al., 2021). According to Rashid et al. (2007), incorporating oats in a diet improves the nutritional value of the diet and its palatability. Ahmed (2013), on the other side, reported that lupine-based dishes were also well accepted in terms of taste score.

**Table 6:** Sensory scores of beverages made from flours of oats, lupine, stinging nettle, and premix.

Run	Components (%)				Sensory Analysis					
	Oat	Lup.	S.N	Prem.	Taste	Appearance	Aroma	Mouth feel	consistency	Overall Acceptance
1	65	10	15	10	3.8 ± 0.13	3.8 ± 0.81	3.2 ± 0.14	3.3 ± 0.11	3.9 ± 0.11	3.7 ± 0.21
2	70	15	5	10	4.2 ± 0.21	4.2 ± 0.55	3.5 ± 0.22	3.6 ± 0.17	4.5 ± 0.23	4.3 ± 0.36
3	62.5	15	12.5	10	3.8 ± 0.31	3.7 ± 0.09	3.3 ± 0.61	3.3 ± 0.52	3.9 ± 0.54	3.6 ± 0.51
4	65	15	10	10	3.7 ± 0.11	3.7 ± 0.14	3.1 ± 1.22	3.0 ± 0.29	4.0 ± 0.12	3.6 ± 0.33
5	62.5	20	7.5	10	3.6 ± 0.09	4.0 ± 0.36	3.0 ± 1.30	3.0 ± 0.13	3.9 ± 0.09	3.7 ± 0.42
6	60	15	15	10	3.6 ± 0.05	3.5 ± 1.42	3.1 ± 1.42	3.2 ± 1.24	3.8 ± 0.08	3.5 ± 0.12
7	65	12.5	12.5	10	3.7 ± 0.12	3.6 ± 0.99	3.1 ± 0.98	3.1 ± 1.13	3.9 ± 0.12	3.8 ± 1.23
8	60	25	5	10	4.5 ± 0.25	4.5 ± 1.30	3.0 ± 0.99	3.1 ± 0.84	3.7 ± 1.30	4.0 ± 0.23
9	70	10	10	10	4.5 ± 0.07	4.2 ± 1.51	3.5 ± 1.21	3.6 ± 0.31	4.5 ± 0.83	4.2 ± 0.12
10	67.5	12.5	10	10	3.7 ± 0.04	3.8 ± 0.84	3.2 ± 0.52	3.5 ± 0.14	4.1 ± 0.21	3.9 ± 0.13
11	67.5	15	7.5	10	3.8 ± 0.01	3.8 ± 0.34	3.3 ± 0.11	3.5 ± 0.34	4.0 ± 0.33	4.0 ± 0.12
	90% oat (control)			10	3.5 ± 0.24	3.6 ± 0.88	3.0 ± 0.90	3.6 ± 0.15	4.2 ± 0.12	3.9 ± 0.07

Values are mean ± standard deviation of 50 rankings on a five-point hedonic scale. Lup: lupine, S.N: stinging nettle, Prem: premix.

The appearance of the beverages ranged from 3.5 to 4.5 and showed significant variation among them. There was a significant difference (p < 0.05) between oat and lupine and the obtained appearance score. Beverages 8 and 6 had the highest and lowest appearance score values, respectively. The appearance score was directly related to the lupine ratio in the blend, which could be due to the appealing yellowish color of lupine flour. Similar to the results of this study, Jayasena and Nasar-Abbas (2012) reported an improvement in pasta color and consumer acceptability due to the inclusion of lupine flour in the blend, which imparted a desirable yellowish color to the pasta. Štefániková et al. (2020) also reported lupine flour was suitable for fortification in various food products. In this study, the green color of stinging nettle flour was balanced by all other composite ingredients. The white color of oat flour, yellowish color of lupines flour, and yellowish color of pumpkin powder in the premix successfully compensated for this limitation.

The mean aroma values of beverages ranged from 3 to 3.5, and there was no statistically significant variation occurred in the aroma score values among the sample beverages (Table 7). The aroma of the beverage was found to be non-significant (p > 0.05) in both linear terms and all the possible interactions. The aroma acceptability of beverages 2 and 9 was the highest, while beverages 5 and 8 were the lowest. The highest mean aroma score was obtained for the beverages with high oat and low lupine; this could be because of the beany flavor of legumes. In support of this argument, Bravo-Núñez and Gómez (2021) reported legume flours could lower the aroma and acceptability. However, according to Schlegel et al. (2021), fermentation is a promising approach for considerably modifying the aroma impressions of legumes.

**Table 7:** Analysis of variance p-value of nutritional composition and sensory properties of the beverage prepared from blends of oat, lupine, and stinging nettle.

Regression Model	Sensory Analysis					
	Taste	Appearance	Aroma	Mouth feel	Consistency	Overall acceptability
Linear^a^	0.021	0.027	0.342	0.345	0.222	0.053
Quadratic^a^	0.021	0.012	0.326	0.397	0.132	0.035
Oat∗Lupine	0.012	0.02	0.214	0.233	0.055	0.032
Oat∗Stinging nettle	0.093	0.147	0.112	0.139	0.077	0.038
Lupine∗Stinging nettle	0.332	0.08	0.991	0.768	0.411	0.123
*R*^2^ (adjusted)	0.77	0.89	0.63	0.53	0.87	0.91

Notes: ^*a*^ Model fitting method used is mixture regression. A regression p-value less than or equal to 0.05 indicates the model explains variation in the response.

The mouthfeel of the beverages ranged from 3 to 3.6, with no significant variation among all the sample beverages. There was no significant difference (p > 0.05) in mouthfeel in both linear terms and all possible interactions. Beverages 2 and 9 received the highest ratings, while beverages 4 and 5 received the lowest. The mouthfeel of fermented beverages improved as the proportion of oats in the blend increased. Food products must have a comfortable mouth feel. An infant, for example, consumes and swallows smooth food rather than a course one (Bazaz et al., 2016).

The results of beverage consistency ranged from 3.7 to 4.5 and showed no significant variation among all the sample beverages. There was no significant difference (p > 0.05) in consistency in both the linear terms and in all the possible interactions. Beverages 2 and 9 were the most consistent, while beverage 8 was the least consistent. The consistency of fermented beverages increased with the ratio of oats in the blend and decreased with the proportion of lupine. Fermentation processes would greatly aid in ensuring consistency and overall sensory properties (Adebo et al., 2017).

The beverages' mean overall acceptability score ranged from 3.5 to 4.3, with a significant difference (p < 0.05) among 11 sample beverages. Beverage 2 had the highest overall acceptability value, while beverage 6 had the lowest. This study revealed that the beverage that was formulated with high oat and low stinging nettle proportions was found to be the most popular. It could be due to oats' high-fat content, which may contribute significantly to the product's palatability and acceptability. Similar to this study, Tiwari et al. (2018) reported that the sensory evaluation of fermented oat spread yielded higher acceptance than unfermented oat spread. In this study, 15% lupine was blended to achieve the best overall sensory acceptance of the final fermented beverage. A similar result was also reported by Yegrem et al. (2021). The blending of 15% lupine with tef flour resulted in the best overall sensory acceptance for a fermented product (*injera*). Aside from blending ratios of ingredients, the food processing method should also be considered to obtain palatable products. The natural fermentation used in this study may also help improve raw cereals' low organoleptic properties. According to Helinck et al. (2004) and Peyer et al. (2016), lactic acid bacteria (LAB), the dominant fermenting microorganisms in spontaneous fermentation, released metabolites such as carboxylic acids and aldehydes, which are reported as the most prominent taste and flavor compounds released during fermentation. In terms of consumer acceptance, these organoleptic properties make fermented beverages more popular than unfermented beverages. Similarly, Schoeman and Manley (2019) and Admassie (2018) demonstrated that traditional processing methods such as roasting and fermentation improve sensory properties by increasing the taste, aroma, texture, and overall acceptability of foods. This study also developed a sensory-acceptable ready-to-drink beverage using traditional processing methods, roasting and fermentation.

### 3.4 Optimization of nutritional and sensory properties

**Figure 1:**
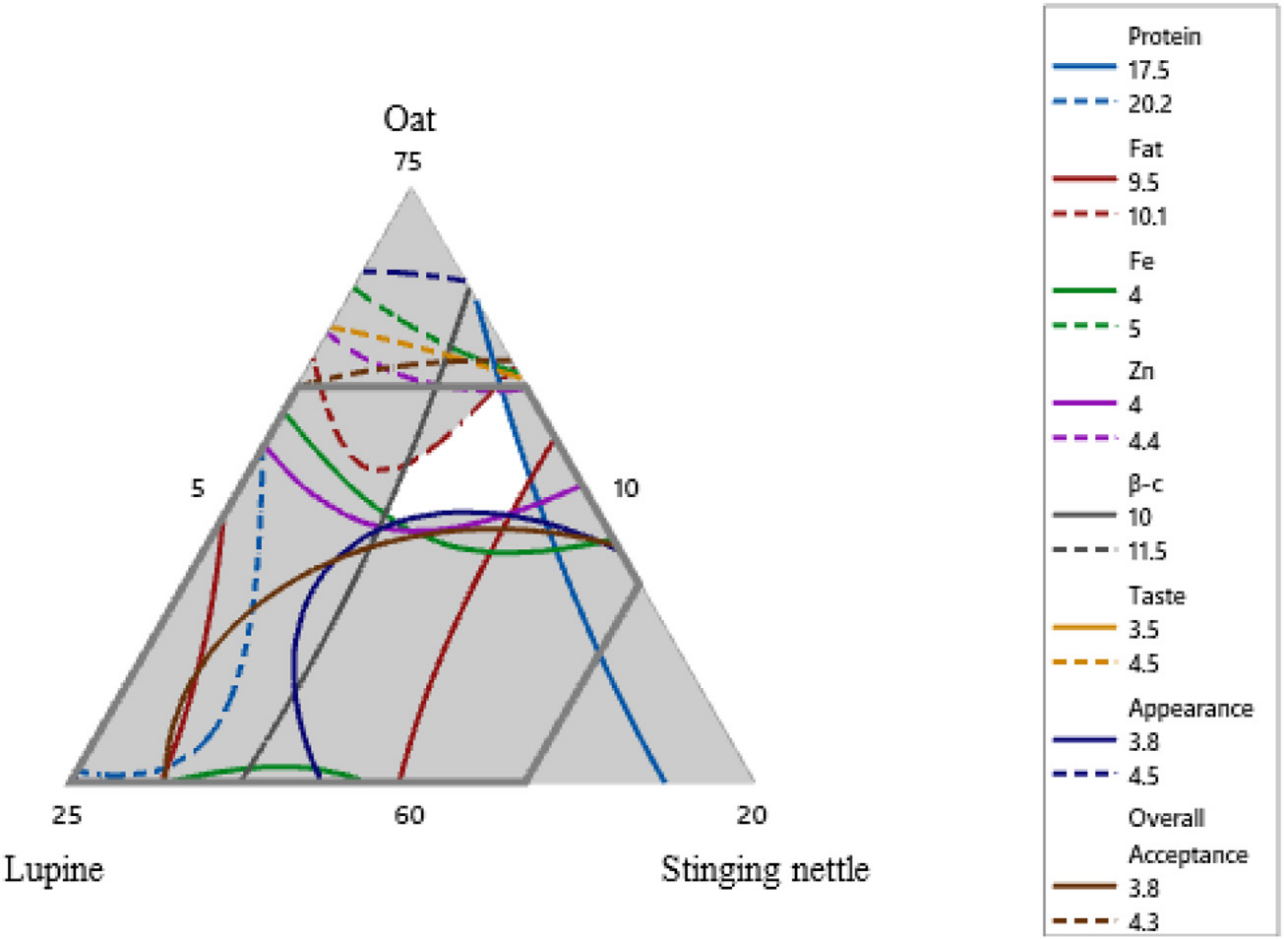
Overlaid contour plots that show the sweet spot for nutritional and sensory analysis. Notes; The white area shows the "sweet spot" that optimizes the response variables listed in the respective legends.

It is necessary to find a balance between nutritional composition and sensory acceptability for the product to reach a larger population. Consumer acceptance of a product is an important factor, especially for a new product business. However, developing a product with high nutritive values and all five sensory qualities that satisfy consumers in most applications is impossible (Mezgebo et al., 2018). In this study, a total of eight responses were optimized to the best optimal range of blending the component flours. For optimization, only responses with a statistically significant relationship to the blend components were taken into account. As a result, the researchers established the following lower and upper limits for responses: protein (17.5–20.2 g/100 g), fat (9.5–10.1 g/100 g), Fe (4–5 mg/100 g), Zn (4–4.4 g/100 g), ß-carotene (10–11.5 μg/g), taste (3.5–4.5), appearance (3.8–4.5), and overall acceptability (3.8–4.3). Figure 1 depicts the superimposed contour plots, with the white area indicating the "sweet spot" where the blending ratio of ingredients results in the best response.

Overall numerical optimization of nutritional and sensory qualities revealed that a blend ratio of 70 g/100 g oats, 11.3 g/100 g lupine, and 8.7 g/100 g stinging nettle resulted in optimal compositions of 18 g/100 g protein, 9.9 g/100 g fat, iron 4.9 mg/100 g, zinc 4.4 mg/100 g, and 9.9 μg/g β-carotene content. Furthermore, this blend received ratings of 4.2, 3.9, and 4 for taste, appearance, and overall acceptability, respectively.

## 4 Conclusion

The current study showed that oat, lupine, stinging nettle, and 10% premix resulted in a nutritionally enhanced beverage with good sensory properties. The study found that increasing the proportion of oat flour in the blend increased fat, carbohydrate, gross energy, and mineral contents. Increasing the proportion of lupine flour in the blend also increased crude protein, crude fiber, and gross energy, while stinging nettle increment in the blend leads to an increase in ash and beta-carotene contents. Except for iron, the molar ratios of anti-nutrients to minerals predict that mineral availability was not suppressed. The best blending ratio (high nutrients and sensory acceptable) was found to be 70% oat, 11.3% lupine, 8.7% stinging nettle, and 10.0% premix. This cereal-legume-vegetable composite beverage is recommended as part of a diet to improve nutritional status in people with limited resources, particularly in low-income countries. Furthermore, the findings also encouraged low-cost, underutilized, and indigenous crops to combat malnutrition.

## Declarations

### Author contribution statement

Getaneh Firew Alemayehu: Conceived and designed the experiments; Performed the experiments; Analyzed and interpreted the data; Contributed reagents, materials, analysis tools or data; Wrote the paper.

Sirawdink Fikreyesus Forsido; Yetenayet B. Tola; Endale Amare: Conceived and designed the experiments; Analyzed and interpreted the data; Wrote the paper.

### Funding statement

This research did not receive any specific grant from funding agencies in the public, commercial, or not-for-profit sectors.

### Data availability statement

The data that has been used is confidential.

### Declaration of interest’s statement

The authors declare no conflict of interest.

### Additional information

No additional information is available for this paper.
